# Parametric Investigation of Particle Swarm Optimization to Improve the Performance of the Adaptive Neuro-Fuzzy Inference System in Determining the Buckling Capacity of Circular Opening Steel Beams

**DOI:** 10.3390/ma13102210

**Published:** 2020-05-12

**Authors:** Quang Hung Nguyen, Hai-Bang Ly, Tien-Thinh Le, Thuy-Anh Nguyen, Viet-Hung Phan, Van Quan Tran, Binh Thai Pham

**Affiliations:** 1 Thuyloi University, Hanoi 100000, Vietnam; 2 University of Transport Technology, Hanoi 100000, Vietnam; anhnt@utt.edu.vn (T.-A.N.); quantv@utt.edu.vn (V.Q.T.); binhpt@utt.edu.vn (B.T.P.); 3 Institute of Research and Development, Duy Tan University, Da Nang 550000, Vietnam; 4 University of Transport and Communications, Ha Noi 100000, Vietnam; phanviethung@utc.edu.vn

**Keywords:** particle swarm parameters, adaptive neuro-fuzzy inference system, circular opening steel beams, buckling capacity

## Abstract

In this paper, the main objectives are to investigate and select the most suitable parameters used in particle swarm optimization (PSO), namely the number of rules (n_rule_), population size (n_pop_), initial weight (w_ini_), personal learning coefficient (c_1_), global learning coefficient (c_2_), and velocity limits (f_v_), in order to improve the performance of the adaptive neuro-fuzzy inference system in determining the buckling capacity of circular opening steel beams. This is an important mechanical property in terms of the safety of structures under subjected loads. An available database of 3645 data samples was used for generation of training (70%) and testing (30%) datasets. Monte Carlo simulations, which are natural variability generators, were used in the training phase of the algorithm. Various statistical measurements, such as root mean square error (RMSE), mean absolute error (MAE), Willmott’s index of agreement (IA), and Pearson’s coefficient of correlation (R), were used to evaluate the performance of the models. The results of the study show that the performance of ANFIS optimized by PSO (ANFIS-PSO) is suitable for determining the buckling capacity of circular opening steel beams, but is very sensitive under different PSO investigation and selection parameters. The findings of this study show that n_rule_ = 10, n_pop_ = 50, w_ini_ = 0.1 to 0.4, c_1_ = [1, 1.4], c_2_ = [1.8, 2], f_v_ = 0.1, which are the most suitable selection values to ensure the best performance for ANFIS-PSO. In short, this study might help in selection of suitable PSO parameters for optimization of the ANFIS model.

## 1. Introduction

Circular opening steel beams have been increasingly acknowledged in structural engineering because of their many remarkable advantages [1], including their ability to bridge the span of a large aperture or their lighter weight compared with conventional steel beams. In general, the industrial approach to producing such a structural member is the rolled method, involving a single steel piece. This is then cut so that the two halves can be assembled, making an I-section, which is also called an H-section steel beam. Hoffman et al. [2] showed that the flexural stiffness and specific gravity per unit length was improved significantly in circular opening steel beams structures. In addition, economic and aesthetics factors are also beneficial points that deserve significant attention [3,4]. A typical structural member has a regular circular openings along its length [1,2,3,4,5,6,7,8], and is about 40–60% deeper and 40–60% stronger than a regular I-section [5,6]. Because of these advantages, circular beams are not only used in lightweight or large-span structures, but are also used for other complex civil engineering structures, such as bridges [9]. Due to the possibility of using circular opening steel beams in various engineering applications, investigation of the failure behavior is crucial to ensure the safety of structures. Several previously published studies on the failure modes of circular beams, for instance the work by Sonck et al. [3], have shown that the web openings are the leading causes of the complex failure behavior of cellular beams, including web post-buckling (WPB), the Vierendeel mechanism (VM), rupture of the web post-weld [1], local web buckling (LWB), and web distortional buckling (WDB) [5,6].

Miscellaneous analysis-related research studies have been conducted to study the behavior of circular opening steel beams [10,11,12], which have mainly focused on the web openings using various numerical approaches [7,9]. As an example, Chung et al. [11] used finite element models with material and geometrical nonlinearity to calculate the behavior of circular beams, resulting in approximately 15.8% of error. Numerical methods help create various case studies in order to gain more knowledge about the working principles of the structures. Taking the work of Panedpojaman and Thepchatri [4] as an example, the authors created a total of 408 nonlinear finite element models using ANSYS software to investigate the behavior of circular steel beams. The results indicated that there is always a small difference between the finite element model and the theoretical formulation. In another study, Sonck et al. [3] generated 597 numerical models, which were calibrated with laboratory tests for 14 geometrically different full-scale steel cellular beams and verified with 1948 numerical analyzes. The results showed that the experimental and numerical curves were identical, with a maximum load gap range of 5.1% to 6.5%. Typically, the numerical models are useful for evaluating the behavior of circular beams [1,3,6,9,13]. However, these model require much effort and the use of modern software and equipment.

Machine learning (ML) algorithms, a branch of artificial intelligence (AI) techniques, have been constantly developed during the past few decades due to the significant increase in computer science [14,15,16,17,18,19,20,21]. Various ML models have been effectively implemented to solve countless specific engineering problems, including in material sciences [22,23,24], geotechnical engineering [25,26,27,28,29], and especially structural engineering [18,30,31,32]. As an example, Vahid et al. [33] selected an artificial neural network (ANN) algorithm, the most popular ML model, to predict the shear capacity of a web opening steel I-beam. The proposed ANN model had better accuracy compared with other existing formulas or theoretical predictions derived from the ACI 318-08 standard. Abambres et al. [34] also used the ANN method to investigate the buckling load capacity of cellular beams under uniformly distributed vertical loads, using eight geometrical parameters. Good results were achieved by the ANN, giving 3.7% for the total error and 0.4% for the average relative error. Blachowski and Pnevmatikos [35] proposed an ANN model for the design and control of the vibration of structural elements under earthquake loading. In the same context of seismic excitation, Pnevmatikos and Thomos [36] employed a stochastic control approach to determine the influence of random characters on the dynamic behavior of engineering structures. The neuro-fuzzy system is another efficient ML algorithm, which has been employed in many structural and material engineering applications, including for steel structures. Seitllari and Naser [37] investigated the performance of an adaptive neuro-fuzzy inference system (ANFIS) in predicting a fire-induced spalling phenomenon in steel-reinforced concrete structures. Naser [38] derived a material model for steel structures, taking into account the dependency of temperature based on machine learning techniques. Basarir et al. [39] compared the performance between conventional regression techniques and ANFIS in predicting the ultimate pure bending of concrete-filled steel tubular members. Naderpour and Mirrashid [40] used ANFIS to predict the shear strength of beams that had been reinforced with steel stirrups. Mermerdaş et al. [41] applied ANFIS to evaluate the flexural behavior of steel circular hollow section (CHS) beams. It was stated that the ANFIS was a promising tool for quick and accurate evaluation of the mechanical behavior of steel-based engineering structures.

In general, the ML algorithms are excellent and effective for evaluating the behavior of structural members, including circular beams. However, their performance depends significantly on the selection of parameters used to learn the models [42]. Therefore, the process of determining such parameters is crucial to obtain highly reliable and accurate prediction results. Concerning the ANN, many parameters could be involved, such as the initial weights, biases to start the training phase, the learning rate, the stopping criterion, the choice of features in the training phase, the choice of the splitting dataset ratio, the number of hidden layers and the corresponding activation functions, the training algorithm, and the number of neurons in each hidden layer [43,44,45]. Considering the ANFIS, two groups of parameters can be considered, namely the nonlinear parameters of the antecedent membership function (MF) and linear parameters of the consequent MF, which depends on the partitioning of the fuzzy space, as well as the type of Sugeno model [46,47]. Besides, many optimization techniques, such as particle swarm optimization (PSO), differential evolution (DE), evolutionary algorithm (EA), genetic algorithm (GA), artificial bee colony (ABC). or cuckoo search (CS) techniques, have been proposed to optimize the parameters of the ML models [48,49]. Each optimization technique also possesses many different parameters that need to be tuned to obtain good prediction performances, inducing the time required to adjust the combination of these parameters [48,49]. Among the well-known optimization techniques, PSO is considered as one of the most popular and effective techniques [50]. Many hybrid ML algorithms have used PSO for the parameter tuning process, including ANN, ANFIS, and Support Vector Machine (SVM) algorithms [51,52,53]. In the literature, limited studies have used ANFIS optimized by PSO (ANFIS-PSO) to predict the mechanical properties of structural members. Moreover, a systematic investigation of ANFIS-PSO parameters under random sampling has not been performed, as the sampling method has been proven to greatly affect the accuracy of the ML algorithms [54].

In this study, the main purpose was to carry out a parametric investigation of PSO parameters to improve the performance of ANFIS in predicting the buckling capacity of circular opening steel beams, which is an important mechanical property that is crucial for the safety of structures under subjected loads. The database used in this work consisted of 3645 data samples, which were derived from numerical results using ANSYS and available in the literature. The parametric studies were carried out with the help of Monte Carlo simulations, which are natural variability generators, in the training phase of the algorithm. Various statistical measurements, such as the root mean square error (RMSE), mean absolute error (MAE), Willmott’s index of agreement (IA), and Pearson’s coefficient of correlation (R), were used to evaluate the performance of the model.

## 2. Novelty and Significance of This Study

As reported in the introduction, the estimation of the buckling capacity of circular opening steel beams is important for the safety of structures under subjected loads. As instability is a complex (nonlinear) problem that is affected by various parameters, the determination of the critical buckling load remains challenge for researchers (engineers) in the fields of mechanics and civil engineering. Despite various experimental works having investigated this problem, it is not easy to derive a generalized expression that considers all the parameters that govern the instability of circular opening steel beams. To overcome this difficulty, the use of ML techniques, such as ANFIS optimized by the PSO algorithm proposed in this study, could be a good choice as a surrogate model. This soft computing method could help to explore the nonlinear relationships between the buckling capacity and the input variables, especially the geometrical parameters of the beams. In addition, the investigation of PSO parameters based on the Monte Carlo random sampling technique could contribute to better knowledge on selection of suitable parameters to achieve better performance with the PSO algorithm, which could be further recommended for other problems. Finally, the proposed ML-based model could be a potential tool for researchers or structural engineers in accurately estimating the buckling capacity of circular opening steel beams, which could (i) work within the ranges of values used in this study for the input variables and (ii) save time and costs in development of other numerical schemes (i.e., finite element models).

## 3. Database Construction

**Figure 1:**
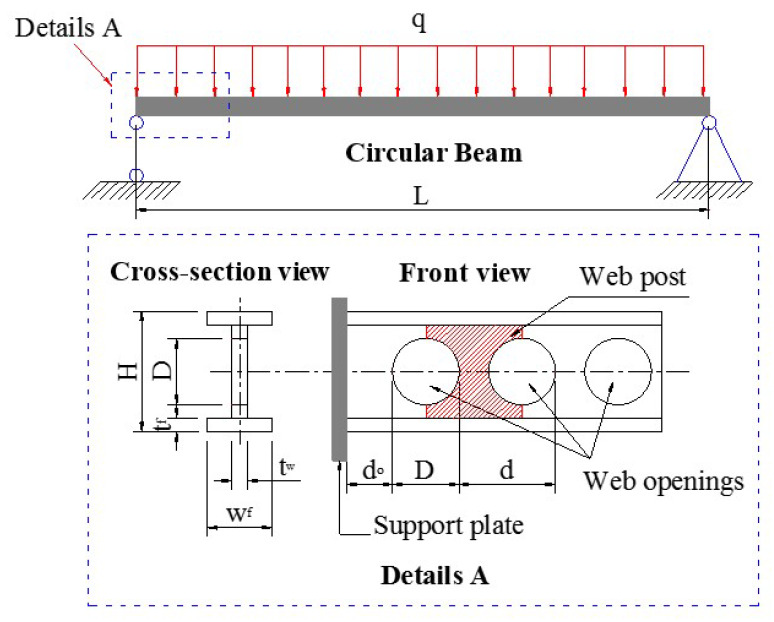
Diagram of circular opening steel beam under uniform loading and its geometrical parameters.

The database in this study was obtained by analyzing 3645 different configurations of circular opening steel beams (Figure 1). It should be noted that the database was extracted from a validated finite element model, which was previously proposed in the literature by Abambres et al. [34]. It consisted of 8 input parameters, namely the length of the beam (denoted as L), the end opening distance (denoted as d_0_), circular opening diameter (denoted as D), the inter-opening distance (denoted as d), the height of the section (denoted as H), the thickness of the web (denoted as t_web_), the width of the flange (denoted as w_flange_), the thickness of the flange (denoted as t_flange_), and the buckling capacity, which was considered as the target variable (denoted as P_u_). It should be pointed out that the database was generated for one material type (with a typical Young’s modulus of 210 GPa and Poisson’s ratio of 0.3). The results of the statistical analysis of the Pu and the corresponding influential parameters are presented in Table 1.

The input and target variables in this work were scaled in the range of [0, 1] to minimize the numerical bias of the dataset. After performing the simulation part, a transformation into the normal range was conducted to better interpret the obtained results. Concerning the development phase, the dataset was split into two parts, namely the training part (70% of the total data) and the testing part (the remaining 30% of the data), which served as the learning and validation phases of the proposed ANFIS-PSO model, respectively.

## 4. Machine Learning Methods

### 4.1. Adaptive Neuro-Fuzzy Inference System

Jang et al. [55] introduced the fuzzy adaptive system of adaptive neurology, called ANFIS, as an improved ML method and a data-driven modeling approach to evaluate the behavior of complex dynamic systems [56,57]. ANFIS aims to systematically generate unknown fuzzy rules from a given set of input and output data. ANFIS creates a functional map that approximates the internal system parameter estimation method [58,59,60]. Fuzzy systems are rule-based systems developed from a set of language rules. These systems can represent any system with good accuracy and are, therefore, considered to be universal approximators. Thus, ANFIS is the most popular neuro-fuzzy hybrid network used for the modeling of complex systems. The ANFIS model’s main strength is that it is a universal approximator with the ability to request interpretable “if–then” rules [61]. In ANFIS, a Sugeno-type fuzzy system was used to construct the five-layer network.

### 4.2. Particle Swarm Optimization (PSO)

Eberhart and Kennedy developed the PSO algorithm in 1995. It is an evolutionary computing technique with a particular enhancement method, population collaboration, and competition based on the simulation of simplified social models, such as bird flocking, fish schooling, and swarming theory [62,63,64,65]. It is a biological-based algorithm that shapes bird flocking social dynamics large number of birds flock synchronously, suddenly change direction, iteratively scatter and group, and eventually perch on a target. The PSO algorithm supports simple rules for bird flocking and acts as an optimizer for nonlinear continuous functions [66]. PSO has gained much attention and has been successfully applied in various fields, especially for unconstrained continuous optimization problems [67]. Indeed, in PSO, a swarm member, also called a particle, is a potential solution, which is used as a search space point. The global equilibrium is known as the food position. The particle has a fitness value and a speed with which to change its flight path for the best swarm experiences to find the global optimum in the D-dimensional solution space. The PSO algorithm is easy to implement and many optimization problems have been empirically shown to perform well [68]. However, its performance depends significantly on the algorithm parameters described below.

#### 4.2.1. Initial Weight (w_ini_)

The particle in the PSO is represented as a real-valued vector containing an instance of all parameters that characterize the problem of optimization. By flying a number of particles, called a swarm, the PSO explores the solution space. The initial swarm is generated at random, and generally consecutive iterations maintain a consistent swarm size. The swarm of particles looks for the optimum target solution in each iteration by referring to past experiences.

#### 4.2.2. Cognition Learning Rate (Personal Learning Coefficient—c_1_)

PSO enriches swarm intelligence by storing the best positions that each particle has visited so far. Particles I recall the best position among those it met, called pbest, and the best positions of its neighbors. There are two variants, namely lbest and gbest, used to hold the neighbors in the best position. The particle in the local version keeps track of the best lbest location obtained by its neighboring local particles. For the global version, any particles in the whole swarm will determine the best location for gbest. Therefore, the gbest model is the lbest model’s special case.

#### 4.2.3. Social Learning Rate (Global Learning Coefficient—c_2_)

PSO starts with the random initialization in the search space of a population (swarm) of individuals (particles) and operates on the particles’ social behavior in the swarm. Consequently, it finds the best global solution by simply adjusting each individual’s trajectory to their own best location and to the best swarm particle in each phase (generation). Nevertheless, the trajectory of each particle in the search space is modified according to their own flying experience and the flying experience of the other particles in the search space by dynamically altering the velocity of each particle.

#### 4.2.4. Number of Particles (Population Size—n_pop_)

The location and speed of the *i*th particle can be expressed in the dimensional search space. Every particle has its own best (pbest) location, according to the best personal objective value at the time t. The world’s best particle (gbest) is the best particle found at time t in the entire swarm.

#### 4.2.5. Velocity Limits (f_v_)

Each particle’s new speed is determined as follows:

(1) $$y_{i,j}(t+1)=wy_{i,j}(t)+c_{1}r_{1}(p_{i,j}-x_{i,j}(t))+c_{2}r_{2}(p_{g,j}-x_{i,j}(t));\;\;\;\;j=1,2,\ldots ,d$$

where c_1_ and c_2_ are constants referred to as acceleration coefficients, w is referred to as the inertia factor, and r_1_ and r_2_ are two independent random numbers distributed evenly within the spectrum. The location of each particle is, thus, modified according to the following equation in each generation:

(2) $$a_{i,j}(t+1)=a_{i,j}(t)+y_{i,j}(t+1),\;\;\;\;\;\;\;\;\;\;j=1,2,3,\ldots ,d$$

In the standard PSO, Equation (1) is used to calculate the new velocity according to its previous velocity and to the distance of its current position from both its own best historical position and its neighbors’ best positions. The value of each factor in Y_i_ can be clamped within the range to monitor excessive particles roaming outside the search area, then the particle flies toward a new location.

### 4.3. Monte Carlo Simulation

The Monte Carlo technique has been commonly used as a variability generator in the training phase of the algorithm, taking into account the randomness of the input space [69,70,71,72]. Hun et al. [73] studied the problem of crack propagation in heterogeneous media within a probabilistic context using Monte Carlo simulations. Additionally, Capillon et al. [74] investigated an uncertainty problem in structural dynamics for composite structures using Monte Carlo simulations. Overall, the Monte Carlo method has been successfully applied to take into account the randomness in the field of mechanics [75,76,77,78,79,80]. The key point of the Monte Carlo method is to repeat the simulations many times to calculate the output responses by randomly choosing values of the input variables in the corresponding space [81,82]. In this manner, all information about the fluctuations in the input space can be transferred to the output response. In this work, a massive numerical parallelization scheme was programmed to conduct the randomness propagation process. The statistical convergence of the Monte Carlo method reflects whether the number of simulations is sufficient, which can be defined as follows [83,84,85]:

(3) $$f_{conv}=\frac{100}{m\underline{S}}\sum_{j=1}^{m}S_{j}$$

where *m* is the number of Monte Carlo iterations, S is the random variable considered, and *S* is the average value of *S*.

### 4.4. Quality Assessment Criteria

In the present work, three quality assessment criteria—the correlation coefficient (R), root mean squared error (RMSE), and mean absolute error (MAE)—have been used in order to validate and test the developed AI models. R^2^ allows us to identify the statistical relationship between two data points and can be calculated using the following equation [86,87,88,89,90,91,92]:

(4) $$R=\sqrt{\frac{\sum_{j=1}^{N}\left(y_{0,j}-\bar{y}\right)\left(y_{p,j}-\bar{y}\right)}{\sqrt{{\sum_{j=1}^{N}\left(y_{0,j}-\bar{y}\right)}^{2}{\sum_{j=1}^{N}\left(y_{p,j}-\bar{y}\right)}^{2}}}}$$

where *N* is the number of observations, *y_p_* and $\bar{y}$ are the predicted and mean predicted values, while *y_0_* and $\bar{y}$ are the measured and mean measured values of Young’s modulus of the nanocomposite, respective *j* = *1*:*N*. In the case of RMSE and MAE, which have the same units as the values being estimated, low value for RMSE and MAE basically indicate good accuracy of the models’ prediction output [93,94]. In an ideal prediction, RMSE and MAE should be zero. RMSE and MAE are given by the following formulae [95,96,97,98,99]:

(5) $$\mathrm{RMSE}=\sqrt{\sum_{i=1}^{N}{\left(y_{0}-y_{p}\right)}^{2}/N}$$

(6) $$\mathrm{MAE}=\frac{1}{N}\sum_{i=1}^{N}\left|y_{0}-y_{p}\right|$$

In addition, the Willmott’s index of agreement (IA) has also been employed in this study. The formulation of IA is given by [100,101]:

(7) $$\mathrm{IA}=1-\frac{\sum_{i=1}^{N}{\left(y_{0}-y_{p}\right)}^{2}}{\sum_{i=1}^{N}{\left(\left|y_{0}-\bar{y}\right|+\left|y_{p}-\bar{y}\right|\right)}^{2}}$$

## 5. Results and Discussion

### 5.1. Description of Parametric Studies

In order to investigate the influence of PSO parameters on the performance of ANFIS, parametric studies were carried out by varying n_rule_, n_pop_, w_ini_, c_1_, c_2_, and f_v_, as indicated in Table 2. It is noteworthy that the proposed range was selected by considering both problem dimensionality (i.e., complexity) and computation time. As recommended by He et al. [102] and Chen et al. [48], the PSO initial weight should be carefully investigated. Therefore, a broad range of w_ini_ was proposed, ranging from 0.1 to 1.2. The number of populations varied from 20 to 300 with a nonconstant step, whereas the coefficients c_1_ and c_2_ ranged from 0.2 to 2 with a resolution of 0.2. The number of fuzzy rules varied from 5 to 40. Finally, the f_v_ ranged from 0.05 to 0.2.

**Figure 2:**
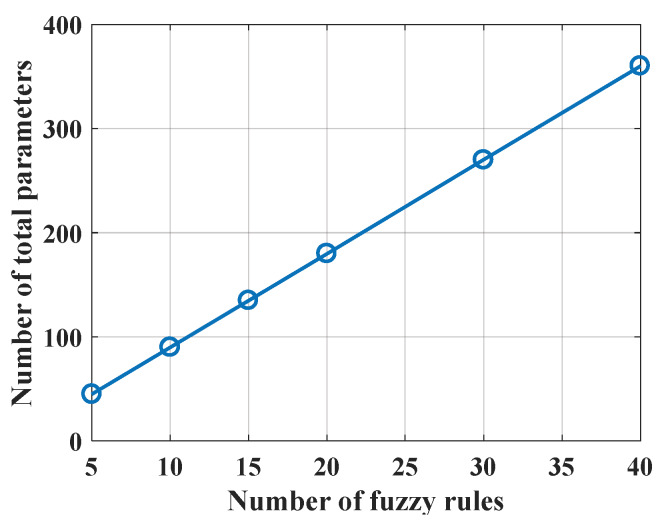
Influence of the number of fuzzy rules on the number of total ANFIS weight parameters to be optimized by PSO.

The relationship between the number of fuzzy rules and the number of total ANFIS weight parameters is depicted in Figure 2. As can be seen, the relationship is linear, showing that as the number of fuzzy rules increases, the number of ANFIS weight parameters increases. For illustration purposes, the number of weight parameters increases from 50 to 370, while the number of fuzzy rules increases from 5 to 40. Additionally, the characteristics of the ANFIS structure are described in Table 3, showing that the Gaussian membership function was used to generate fuzzy rules.

### 5.2. Preliminary Analyses

#### 5.2.1. Computation Time

**Figure 3:**
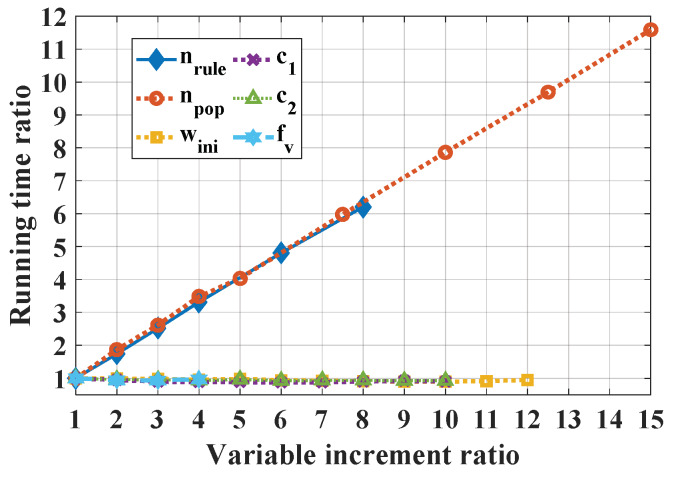
Influence of variable increment ratio on running time, noting that both n_rule_ and n_pop_ exhibit a slope coefficient of 0.75.

Figure 3 presents the influence of n_rule_ and swarm parameters on the computation time. It is worth noting that the running time was scaled with respect to the minimum value of the corresponding parameter. For instance, the computation time using n_rule_ = 10 is two times larger than the case using n_rule_ = 5. Additionally, in Figure 3, it is seen that n_rule_ and n_pop_ exhibited the highest slope (about 0.75), confirming that these two parameters required considerable computation time. For all other parameters, the computation time remained constant when increasing the value of the parameter.

#### 5.2.2. PSO Stopping Criterion

**Figure 4:**
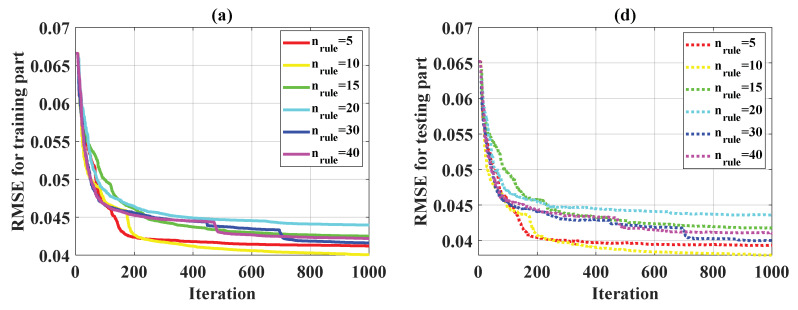

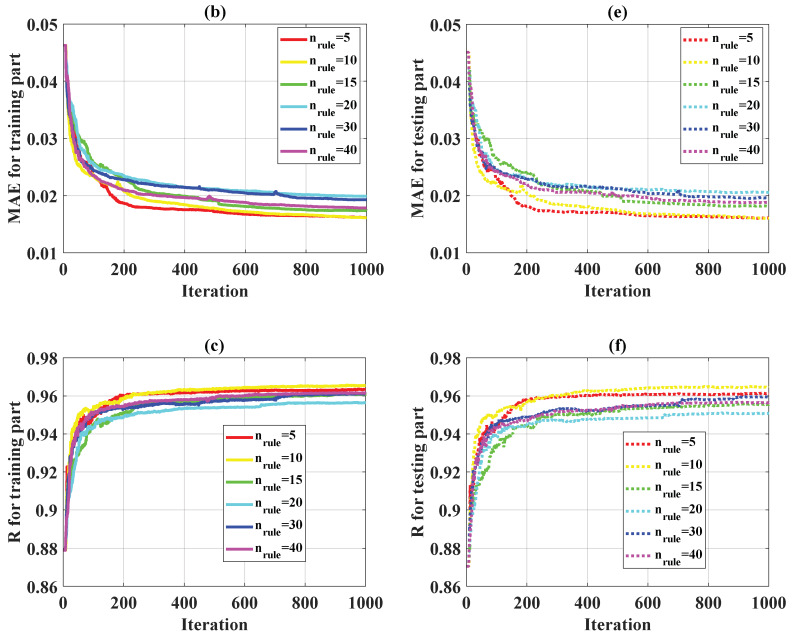
Convergence of several statistical criteria over 1000 iterations in terms of n_rule_ for the training part: (**a**) RMSE, (**b**) MAE, (**c**) R. Convergence of several statistical criteria over 1000 iterations in terms of n_rule_ for the testing part: (**d**) RMSE, (**e**) MAE, (**f**) R.

**Figure 5:**
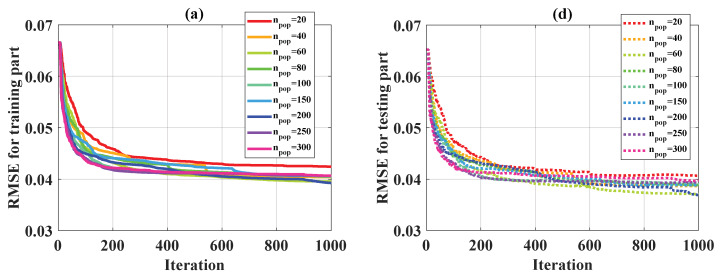

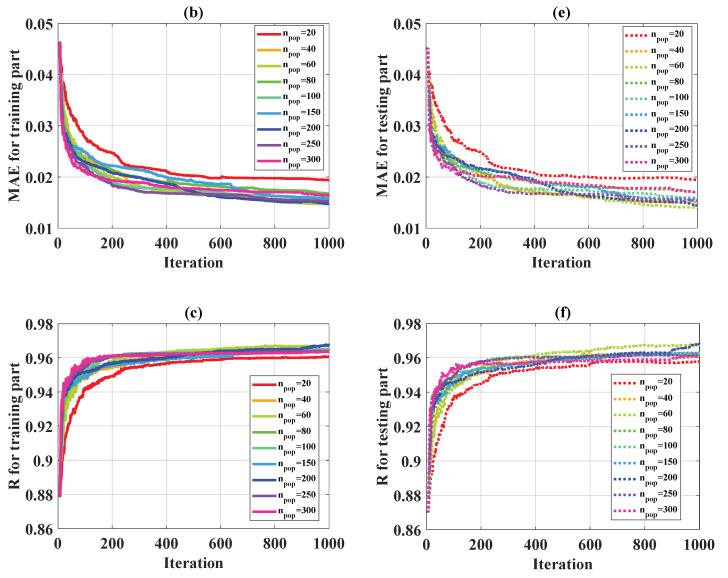
Convergence of several statistical criteria over 1000 iterations in terms of n_pop_ for the training part: (**a**) RMSE, (**b**) MAE, (**c**) R. Convergence of several statistical criteria over 1000 iterations in terms of n_pop_ for the testing part: (**d**) RMSE, (**e**) MAE, (**f**) R.

In this study, 1000 iterations were applied as a stopping criterion in the optimization problem for the weight parameters of ANFIS. Figure 4 shows the convergence of statistical criteria in the function of n_rule_, whereas Figure 5 presents the convergence of these criteria regarding n_pop_. For the evaluation of RMSE, MAE, and R over 1000 iterations in 6 cases for different n_rule_, the training parts are given in Figure 4a–c, whereas the testing parts are displayed in Figure 4d–f. It was observed that at least 800 iterations were required to obtain convergence results for RMSE, MAE, and R for all the cases. However, no specific trend could be deduced in order to obtain the best n_rule_ parameter. Finally, it is worth noting that for all the cases of n_rule_, the values of RMSE, MAE, and R for the testing part were very close. Indeed, the values of RMSE for the testing part ranged from 0.038 to 0.043, the values of MAE for the testing part varied from 0.015 to 0.022, and those of R ranged from 0.95 to 0.97. The evaluation of RMSE, MAE, R over 1000 iterations in 9 cases of n_pope_ is shown (Figure 5). Similar results were obtained as for n_rule_. At least 800 iterations were needed to obtain the convergence results.

#### 5.2.3. Statistical Convergence

**Figure 6:**
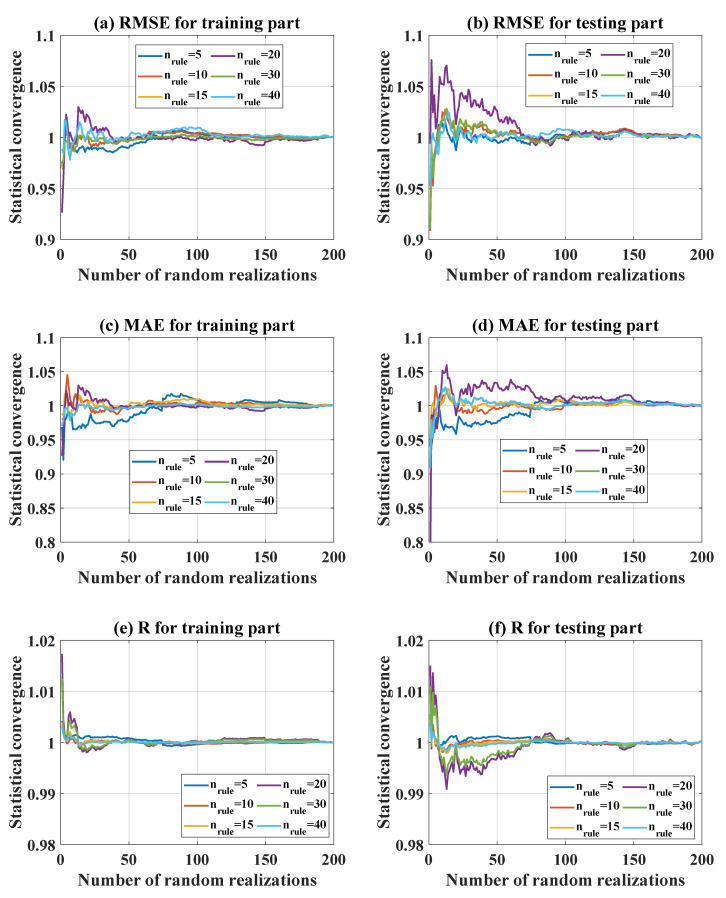
Statistical convergence over 200 random realizations in terms of n_rule_ for the training part: (**a**) RMSE, (**b**) MAE, (**c**) R. Statistical convergence over 200 random realizations in terms of n_rule_ for the testing part: (**d**) RMSE, (**e**) MAE, (**f**) R.

**Figure 7:**
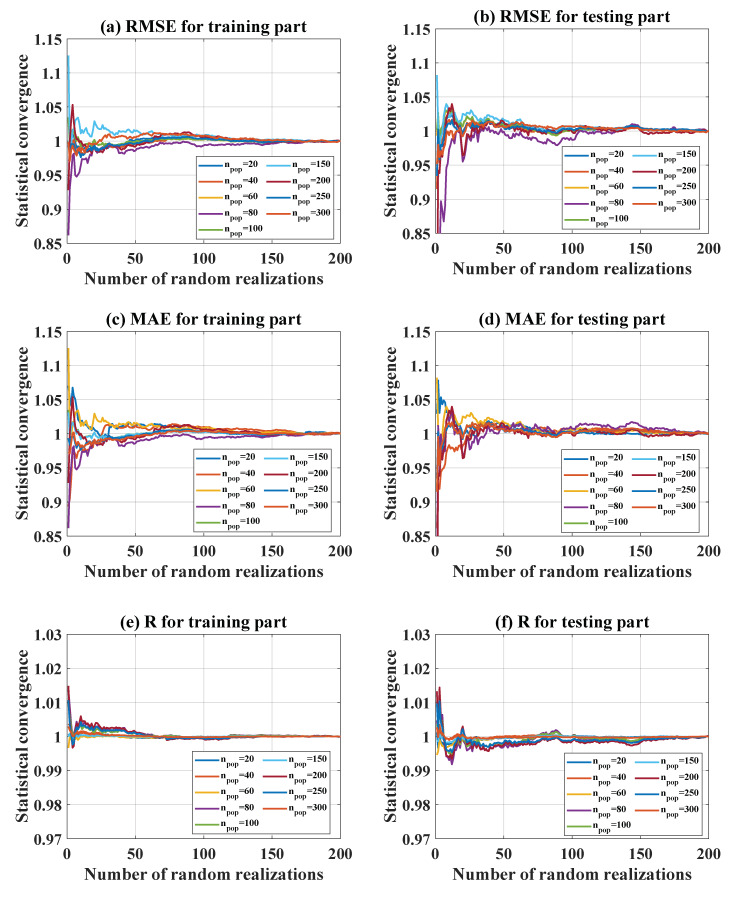
Statistical convergence over 200 random realizations in terms of n_pop_ for the training part: (**a**) RMSE, (**b**) MAE, (**c**) R. Statistical convergence over 200 random realizations in terms of n_pop_ for the testing part: (**d**) RMSE, (**e**) MAE, (**f**) R.

In order to take into account variability in the input space, 200 random realizations were performed for each configuration. These realizations increased the influence of the probability density function of inputs on the optimization results. In terms of n_rule_, Figure 6a–c indicate the statistical convergence of RMSE, MAE, and R for the training part, whereas Figure 6d–f present the statistical convergence of the same parameters for the testing part, respectively. It can be seen that after about 100 random realizations, statistical convergence was reached, which was correct for all the tested cases. Similarly, Figure 7 shows the statistical convergence in terms of n_pop_ for both training and testing parts. Similarly, 200 random realizations were observed to be sufficient to achieve reliable results.

### 5.3. Parametric Performance

#### 5.3.1. Influence of Number of Rules (n_rule_)

**Figure 8:**
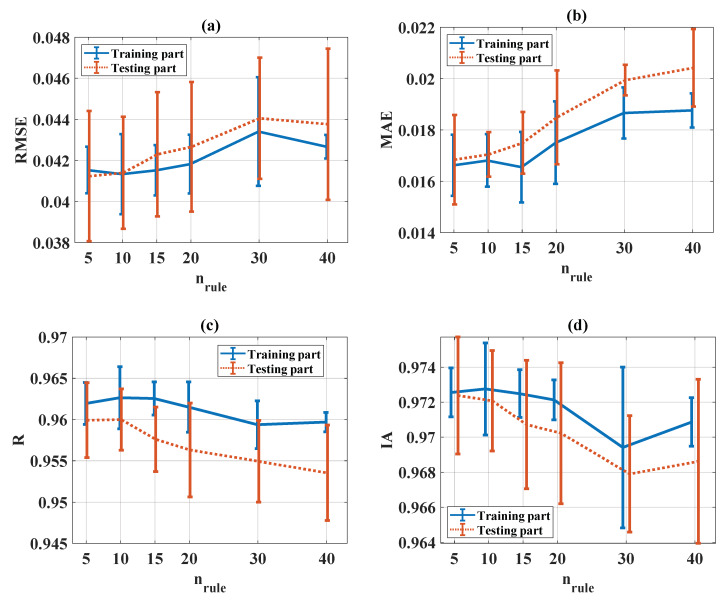
Evaluation of statistical criteria in the function of n_rule_: (**a**) RMSE, (**b**) MAE, (**c**) R, and (**d**) IA.

The evaluation of RMSE, MAE, R, and IA in the function of n_rule_ is presented in Figure 8a–d, respectively, for both training and testing parts. It can be seen that the accuracy of the ANFIS-PSO reduced when the number of n_rule_ increased (i.e., RMSE and MAE increased, while R and IA decreased). It is worth noting that the higher the number of rules, the larger dimensionality of the problem (Figure 2). Therefore, regarding the total number of ANFIS weight parameters, the computation time, and the average value of the statistical criteria (RMSE, MAE, R, and IA), n_rule_ = 10 was considered as the most appropriate value.

#### 5.3.2. Influence of Population Number (n_pop_)

**Figure 9:**
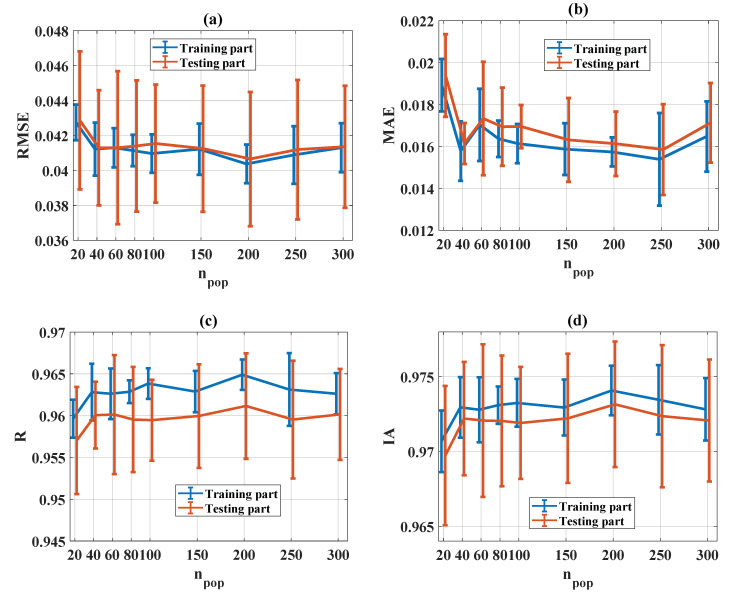
Evaluation of statistical criteria in the function of n_pop_: (**a**) RMSE, (**b**) MAE, (**c**) R, and (**d**) IA.

The evaluation of statistical criteria in the function of n_pop_ for RMSE, MAE, R, and IA is shown in Figure 9a–d, respectively, for both training and testing parts. It can be seen that except for the low value for population size (i.e., n_pop_ = 20), all other n_pop_ values show good prediction results, especially for n_pop_ = 200. However, as introduced in the preliminary analyses for computation time, the higher the number of n_pop_, the more time is consumed. Finally, n_pop_ = 50 was chosen as the most appropriate average value for statistical criteria and computation time.

#### 5.3.3. Influence of Initial Weight (w_ini_)

**Figure 10:**
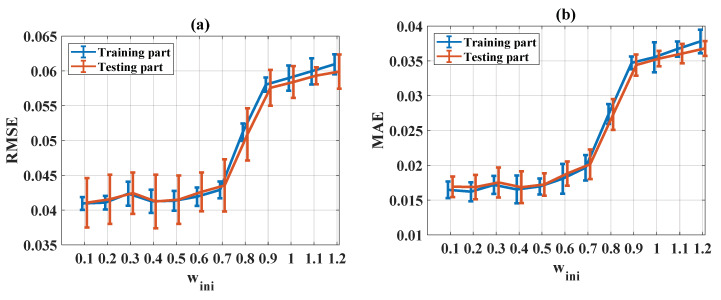

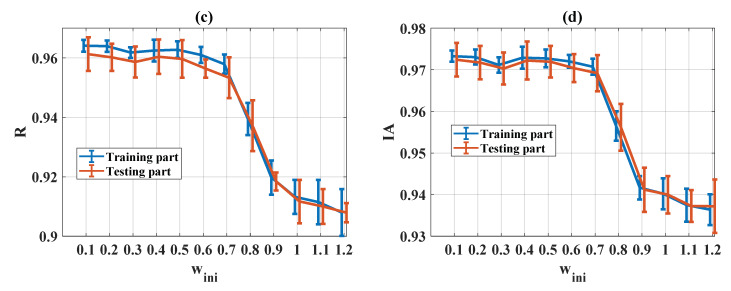
Evaluation of statistical criteria in the function of w_ini_: (**a**) RMSE, (**b**) MAE, (**c**) R, and (**d**) IA.

The evaluation of statistical criteria in the function of w_ini_ for RMSE, MAE, R, and IA is shown in Figure 10a–d, respectively, for both training and testing parts. It can be seen that poor prediction performance was obtained when w_ini_ was larger than 0.5 (i.e., an increase of RMSE and MAE values and a decrease of R and IA values). Regarding the statistical criteria (RMSE, MAE, R, and IA), a w_ini_ value range of between 0.1 and 0.4 was the most appropriate.

#### 5.3.4. Influence of Personal Learning Coefficient (c_1_)

**Figure 11:**
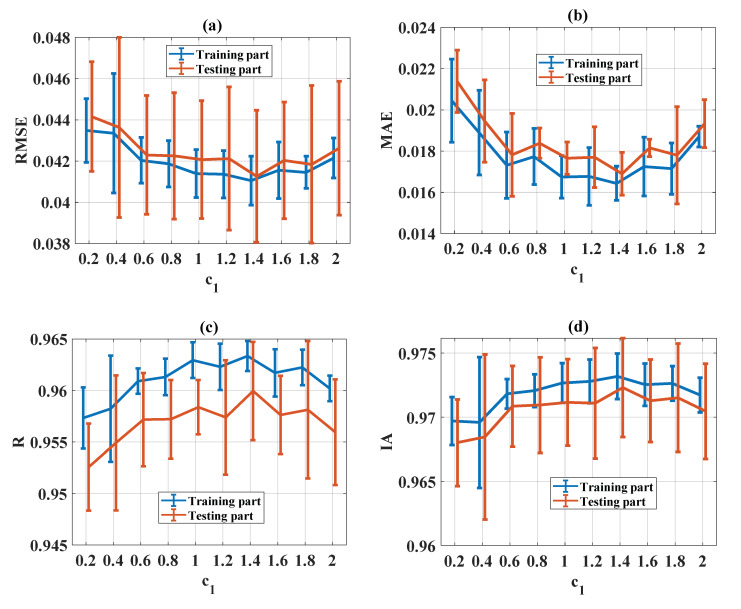
Evaluation of statistical criteria in the function of c_1_: (**a**) RMSE, (**b**) MAE, (**c**) R, and (**d**) IA.

The evaluation of statistical criteria in the function of c_1_ for RMSE, MAE, R, and IA is shown in Figure 11a–d, respectively, for both training and testing parts. It can be seen that good prediction performance was obtained when c_1_ was in the range of [1, 1.4] for all statistical criteria (RMSE, MAE, R, and IA). Therefore, c_1_ = [1, 1.4] was the most appropriate value.

#### 5.3.5. Influence of Global Learning Coefficient

**Figure 12:**
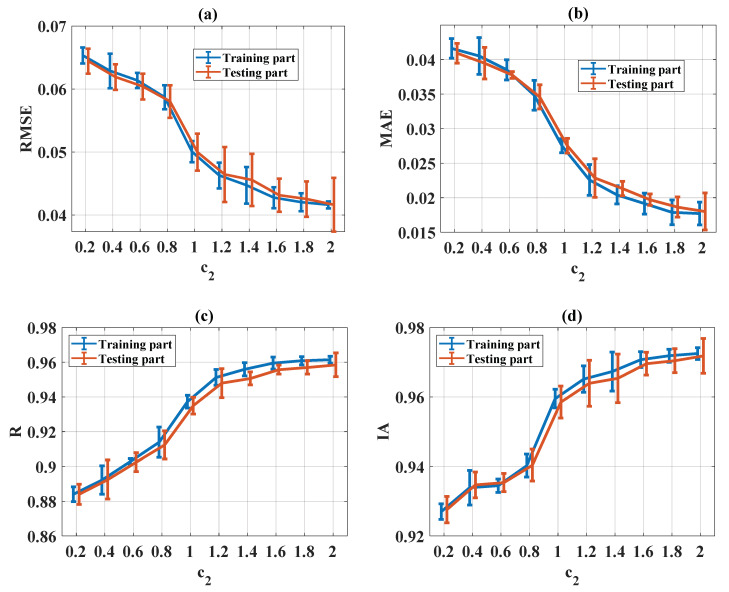
Evaluation of statistical criteria in the function of c_2_: (**a**) RMSE, (**b**) MAE, (**c**) R, and (**d**) IA.

The evaluation of statistical criteria in the function of c_2_ for RMSE, MAE, R, and IA is shown in Figure 12a–d, respectively, for both training and testing parts. It can be seen that good prediction performance was obtained when c_2_ was higher than 1.8 for all statistical criteria (RMSE, MAE, R, and IA). Therefore, c_2_ = [1.8, 2] was the most appropriate value.

#### 5.3.6. Influence of Velocity Limits

**Figure 13:**
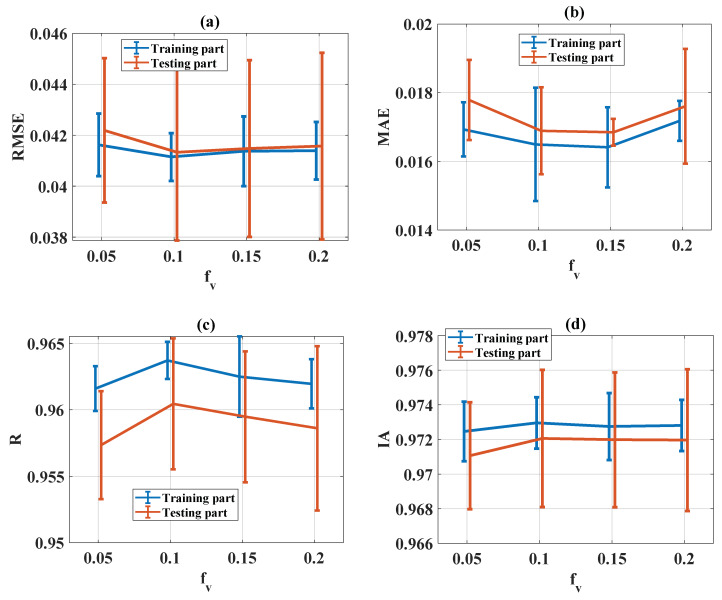
Evaluation of statistical criteria in the function of f_v_: (**a**) RMSE, (**b**) MAE, (**c**) R and (**d**) IA.

The evaluation of statistical criteria in the function of f_v_ for RMSE, MAE, R, and IA is shown in Figure 13a–d, respectively, for both training and testing parts. It can be seen that no influence could be established regarding all statistical criteria (RMSE, MAE, R, and IA). Therefore, f_v_ = 0.1 was finally chosen.

### 5.4. Prediction Capability of the ANFIS-PSO Model using Optimal Configuration

**Figure 14:**
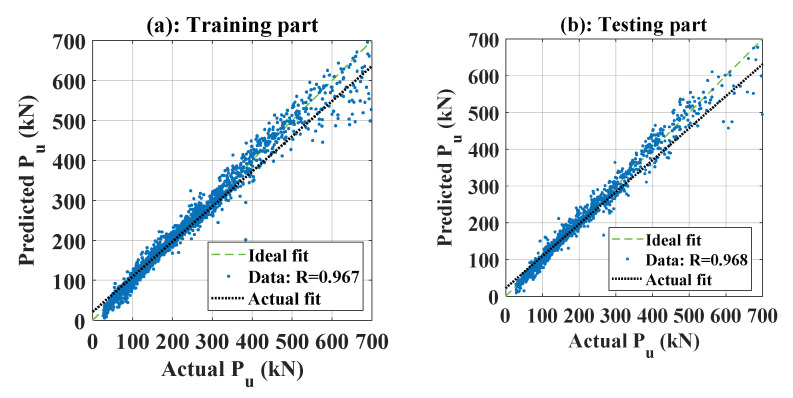
Graphs of regression plots between actual and predicted P_u_ (kN) for the (**a**) training part and (**b**) testing part.

**Figure 15:**
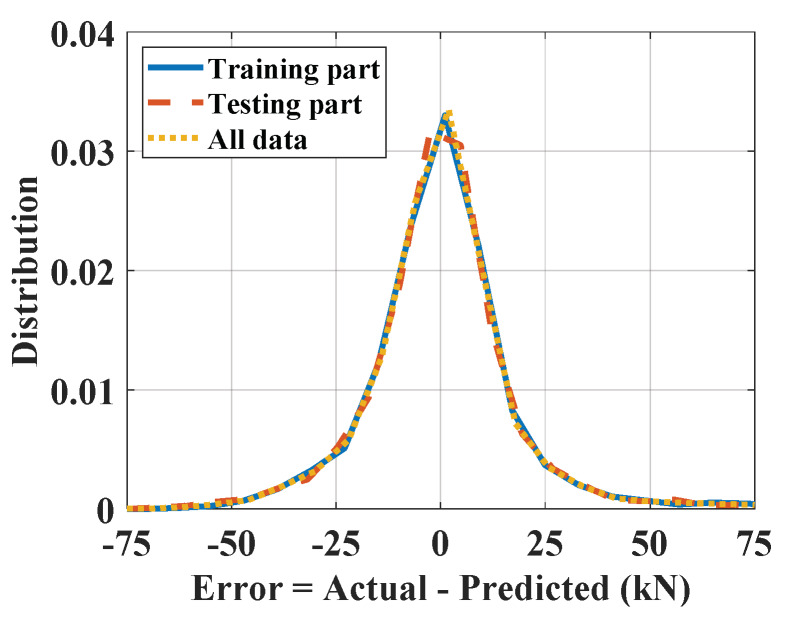
Distribution of errors.

Table 4 summarizes all of the optimal values, as identified previously. By using the optimal coefficient in Table 4, a regression graph between the real and predicted P_u_ (kN) is shown in Figure 14. The slope of the ideal fit was then used to measure the angle between the *x*-axis and the ideal fit, with angles closer than 45° showing better performance. Figure 14a shows the predictability when using the training set, whereas Figure 14b shows the same information applied to the testing set. In both cases, the angles generated by the predicted output had slopes close to that of the ideal fit. This showed that the performance of the proposed model was consistent. Figure 15 shows the error distribution graph using the training part, testing part, and all data. In short, using the selected number of fuzzy rules and PSO parameters, the prediction model gave excellent results (Table 5).

### 5.5. Sensitivity Analysis

**Figure 16:**
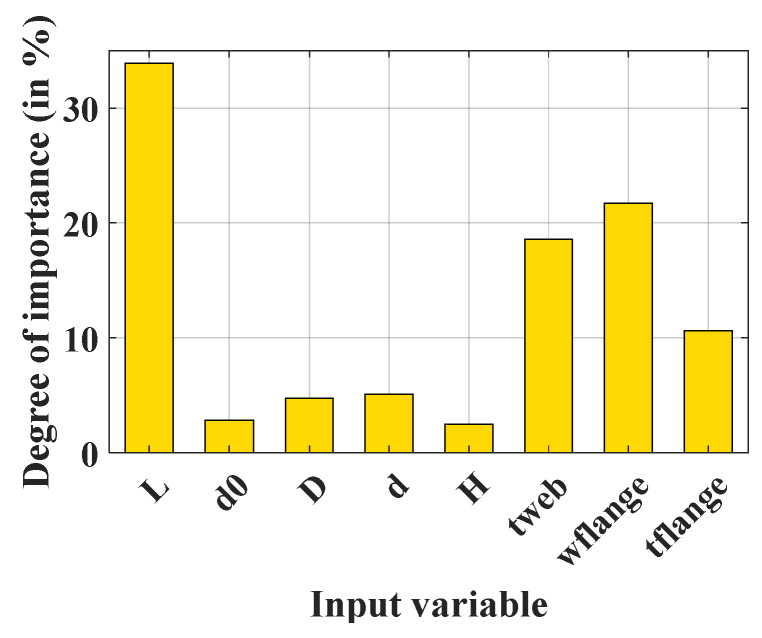
Bar graph showing the estimations of degree of importance values.

The sensitivity analysis was performed in order to explore the degree of importance of each input variable using the ANFIS-PSO model. For this, quantile values at 21 points (from 0% to 100%, with a step of 5%) of each input variable were collected from the database and served as a new dataset for the calculation of critical buckling load. More precisely, for a given input, its value varied from 0% to 100%, while all other inputs remained at their median (50%). This variation of values following the probability distribution allows the influence of each input variable to be explored based on their statistical behavior. The results of the sensitivity analysis are indicated in Figure 16 in a bar graph (scaled into the range of 0% to 100%). It can be seen that all variables influenced the prediction of critical buckling load through the ANFIS-PSO model. The most important input variables were L, w_flange_, t_web_, and t_flange_, which gave degree of importance values of 33.9%, 21.7%, 18.6%, and 10.6%, respectively. This information is strongly relevant and in good agreement with the literature, in which the length of the beam and geometrical parameter of the cross-section are the most important parameters [3,4,5]. However, it can be seen in Figure 16 that the height of the beam does not seriously affect the buckling capacity of the structural members. It should be noted that only three independent values of the section’s height were used to generate the database; for example, 420, 560, and 700 mm. Consequently, the linear correlation coefficient between the section’s height and the buckling capacity was only −0.092. On the contrary, the minimum value of the beam’s length was 4000 mm (approximately 5.7 times larger than the maximum section’s height) and five independent values were used to generate the database, ranging from 4000 to 8000 mm, with a step of 1000 mm. Thus, the linear correlation coefficient between the beam’s length and the buckling capacity was −0.667 (approximately 7.25 times bigger than the linear correlation coefficient between the section’s height and the buckling capacity). Consequently, a larger database should be considered in future studies to estimate the degree of importance of the section’s height.

**Figure 17:**
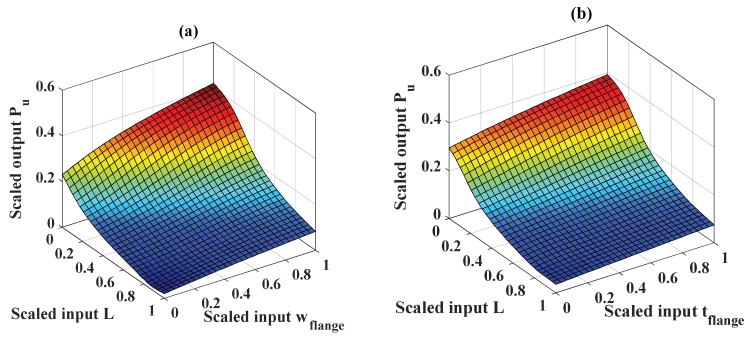

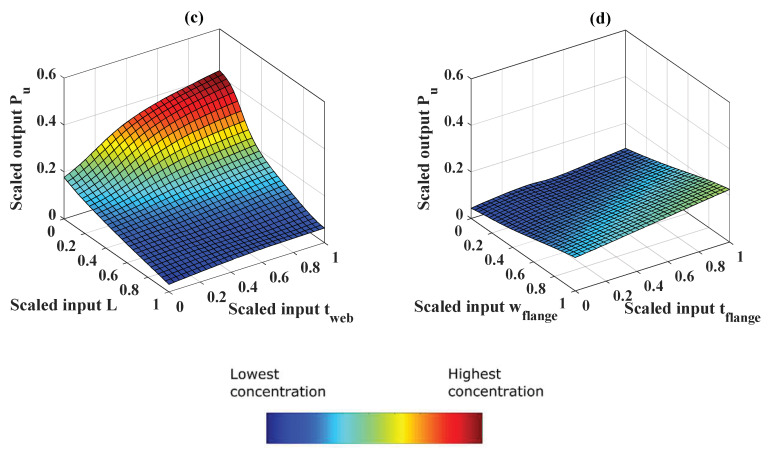
Three-dimensional scaled input–output maps: (**a**) L-w_flange_, (**b**) L-t_flange_, (**c**) L-t_web_, and (**d**) w_flange_-t_flange_.

The sensitivity analysis presented above demonstrates that the ML technique could assist in the design phase for circular opening steel beams. In addition to reliable prediction of the critical buckling load, the ANFIS-PSO model can also assist in the creation of input–output maps, as illustrated in Figure 17. In particular, as L, t_web_, w_flange_, and t_flange_ were the most important variables, they are used for map illustrations in this section. The values of the remaining variables were kept constant. In Figure 17, four maps of critical buckling load are presented (with the same color range), involving the relationship between P_u_ and L-w_flange_, L-t_flange_, L-t_web_, and w_flange_-t_flange_, respectively. As can be seen from the surface plots, the input–output relationship exhibited nonlinear behavior, which cannot be easily identified from the database. Figure 17a shows that a maximum value for the critical buckling load can be obtained if L reaches its minimum and w_flange_ reaches its maximum value. On the other hand, the critical buckling load reaches its minimum if L reaches its highest value and w_flange_ reaches its lowest value. This map confirms the negative effect of L, as pointed out in the literature [4]. In Figure 17b,c, the same results are obtained as in Figure 17a. This observation again confirms that the geometrical parameters of the cross-section are highly important [1,5]. Such quantitative information allows the design and analysis recommendations to be explored, as well as for new beam configurations to be generated (within the range of variables considered in this present study).

## 6. Conclusions

PSO is one of the most popular optimization techniques used to optimize and improve the performance of machine learning models in terms of classification and regression. However, its effectiveness depends significantly on the selection of parameters used to train this technique. In this paper, investigation and selection of PSO parameters was carried out to improve and optimize the performance of the ANFIS model, which is one of the most popular and effective ML models, for prediction of the buckling capacity of circular opening steel beams. Different parameters (n_rule_, n_pop_, w_ini_, c_1_, c_2_, and f_v_) of PSO were tuned on 3645 available data samples to determine the best values for optimization of the performance of ANFIS.

The results show that the performance of ANFIS optimized by PSO (ANFIS-PSO) is suitable for determining the buckling capacity of circular opening steel beams, but is very sensitive under different PSO investigation and selection parameters. The results also show that n_rule_ = 10, n_pop_ = 50, w_ini_ = 0.1 to 0.4, c_1_ = [1, 1.4], c_2_ = [1.8, 2], and f_v_ = 0.1 are the most suitable selection settings in order to get the best performance from ANFIS-PSO. The sensitivity analysis shows that L, w_flange_, t_web_, and t_flange_ are the most important input variables used for prediction of the buckling capacity of circular opening steel beams.

In short, this study might help in selection of the suitable PSO parameters for optimization of ANFIS in determining the buckling capacity of circular opening steel beams. It also helps in suitable selection of input variables for better prediction of the buckling capacity of circular opening steel beams. However, it is noted that the optimal values of PSO parameters found in this study are suitable for the ANFIS model in determining the buckling capacity of circular opening steel beams. Thus, it is suggested that these parameters should be validated with other ML models applied in other problems. Finally, variation in the mechanical properties of material used should be investigated in further research, as this is important from a physics perspective.

## Figures and Tables

**Table 1:** Initial statistical analysis of the dataset.

Variable	Length of Beam	End Opening Distance	Opening Diameter	Inter-Opening Distance	Height of Section	Thickness of Web	Width of Flange	Thickness of Flange	Buckling Capacity
Symbol	L	d_0_	D	d	H	t_web_	w_flange_	t_flange_	P_u_
Unit	m	mm	mm	mm	mm	mm	mm	mm	N/m
Role	Input	Input	Input	Input	Input	Input	Input	Input	Output
Min	4.0	12.0	247.0	24.70	420.00	9.0	162.0	15.0	26.4
MD ^a^	6.0	256.5	373.0	108.17	560.00	12.0	216.0	20.0	169.3
Max	8.0	718.0	560.0	274.40	700.00	15.0	270.0	25.0	1361.7
Mean	6.0	265.4	383.6	112.51	560.00	12.0	216.0	20.0	225.7
SD ^b^	1.4	157.5	93.0	68.51	114.33	2.5	44.1	4.1	182.5
CV ^c^	23.6	59.3	24.2	60.90	20.42	20.4	20.4	20.4	80.9

^a^ Median. ^b^ Standard deviation. ^c^ Coefficient of variation (%).

**Table 2:** Values used for parameters in parametric studies.

Parameters	Values Used											
n_rule_	5	10	15	20	30	40						
n_pop_	20	40	60	80	100	150	200	250	300			
w_ini_	0.1	0.2	0.3	0.4	0.5	0.6	0.7	0.8	0.9	1	1.1	1.2
c_1_	0.2	0.4	0.6	0.8	1	1.2	1.4	1.6	1.8	2		
c_2_	0.2	0.4	0.6	0.8	1	1.2	1.4	1.6	1.8	2		
f_v_	0.05	0.1	0.15	0.2								

**Table 3:** Characteristics of ANFIS structure.

Parameter	Description
Number of inputs	8
Number of outputs	1
Input membership function type	Gaussian
Number of parameter per membership function	2
Number of fuzzy rules	n_rule_
Output membership function type	Linear
Number of nonlinear parameters	8 × 2 × n_rule_
Number of linear parameters	9 × n_rule_
Number of total parameters	25 × n_rule_

**Table 4:** Parameters used as optimum.

Parameter	Optimal Value	Final Selection
n_rule_	10	10
n_pop_	50	50
w_ini_	0.1–0.4	0.4
c_1_	1–1.4	1
c_2_	1.8–2	2
f_v_	0.1	0.1

**Table 5:** Prediction capability.

Part	RMSE	MAE	R	IA	Error Mean	Error Std	Running Time
Training	0.040	0.015	0.967	0.976	0.006	0.039	8 min
Testing	0.037	0.014	0.968	0.977	0.005	0.037	

## Abbreviations

**Symbol**	**Explanation**	**SI Unit**
ANFIS	Adaptive neuro-fuzzy inference system	
PSO	Particle swarm optimization	
R	Correlation coefficient	
RMSE	Root mean squared error	
MAE	Mean absolute error	
IA	Index of agreement	
CV	Coefficient of variation	%
n_rule_	Number of fuzzy rules	
n_pop_	PSO population size	
w_ini_	PSO initial weight	
c_1_	PSO cognition learning rate	
c_2_	PSO social learning rate	
f_v_	PSO velocity limits	
